# *N*-Cyclo­hexyl-3,4,5-trimethoxy­benzamide

**DOI:** 10.1107/S1600536809050284

**Published:** 2009-11-28

**Authors:** Aamer Saeed, Muhammad Arshad, Rasheed Ahmad Khera, Michael Bolte

**Affiliations:** a Department of Chemistry, Quaid-i-Azam University, Islamabad 45320, Pakistan; b Chemistry Division, Directorate of Science, PINSTECH, Nilore, Islamabad, Pakistan; c Institut für Anorganische Chemie, J. W. Goethe-Universität Frankfurt, Max-von-Laue-Strasse 7, 60438 Frankfurt/Main, Germany

## Abstract

The 3,5-meth­oxy groups in the title compound, C_16_H_23_NO_4_, are almost coplanar with the aromatic ring, whereas the 4-meth­oxy group is bent out of this plane. The three CH_3_—O—C—C torsion angles are −1.51 (18), 0.73 (19) and 75.33 (15)°. The cyclo­hexane ring adopts a chair conformation. In the crystal, mol­ecules are connected by inter­molecular N—H⋯O hydrogen bonds into chains running along the *b* axis.

## Related literature

For the biological activity of benzanilides, see: Olsson *et al.* (2002 ▶); Lindgren *et al.* (2001 ▶); Calderone *et al.* (2006 ▶). For the use of benzamides in organic synthesis, see: Zhichkin *et al.* (2007 ▶); Beccalli *et al.* (2005 ▶). For related structures, see: Bowes *et al.* (2003 ▶); Chopra & Guru Row (2008 ▶); Kashino *et al.* (1979 ▶); Saeed *et al.* (2008 ▶).
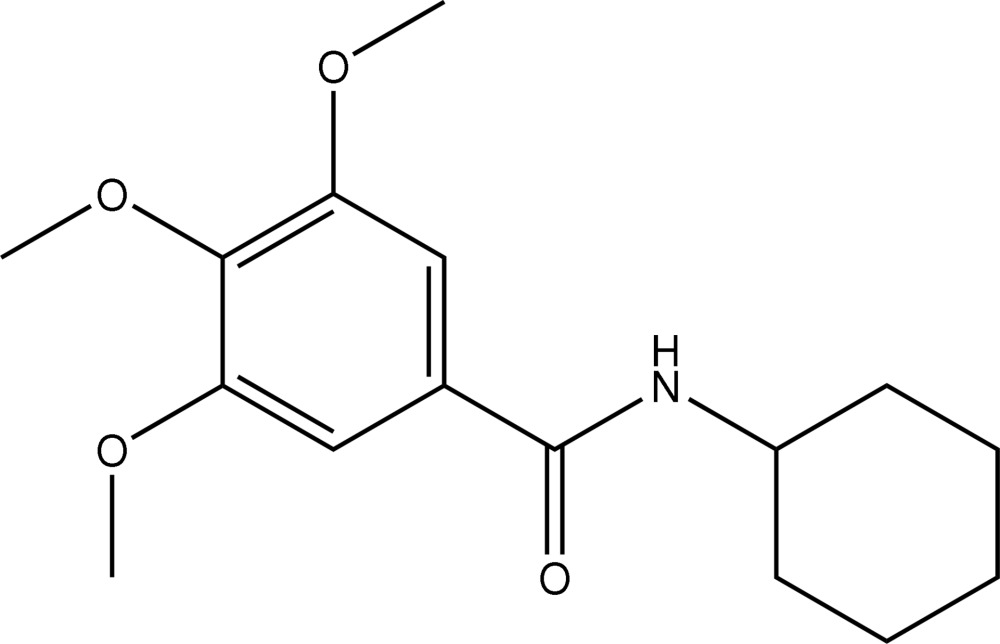


## Experimental

### 

#### Crystal data

C_16_H_23_NO_4_
*M* *_r_* = 293.35
Monoclinic, 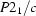

*a* = 23.4539 (19) Å
*b* = 5.2145 (6) Å
*c* = 12.4559 (10) Å
β = 92.886 (6)°
*V* = 1521.4 (2) Å^3^
*Z* = 4
Mo *K*α radiation
μ = 0.09 mm^−1^
*T* = 173 K
0.37 × 0.37 × 0.33 mm

#### Data collection

Stoe IPDSII two-circle diffractometer
Absorption correction: none
6868 measured reflections
2823 independent reflections
2360 reflections with *I* > 2σ(*I*)
*R*_int_ = 0.062

#### Refinement

*R*[*F* ^2^ > 2σ(*F* ^2^)] = 0.040
*wR*(*F* ^2^) = 0.109
*S* = 1.05
2823 reflections
198 parameters
H atoms treated by a mixture of independent and constrained refinement
Δρ_max_ = 0.27 e Å^−3^
Δρ_min_ = −0.22 e Å^−3^

### 

Data collection: *X-AREA* (Stoe & Cie, 2001 ▶); cell refinement: *X-AREA*; data reduction: *X-AREA*; program(s) used to solve structure: *SHELXS97* (Sheldrick, 2008 ▶); program(s) used to refine structure: *SHELXL97* (Sheldrick, 2008 ▶); molecular graphics: *XP* in *SHELXTL* (Sheldrick, 2008 ▶); software used to prepare material for publication: *PLATON* (Spek, 2009 ▶) and *SHELXL97*.

## Supplementary Material

Crystal structure: contains datablocks global, I. DOI: 10.1107/S1600536809050284/fj2261sup1.cif

Structure factors: contains datablocks I. DOI: 10.1107/S1600536809050284/fj2261Isup2.hkl

Additional supplementary materials:  crystallographic information; 3D view; checkCIF report

## Figures and Tables

**Table 1:** Hydrogen-bond geometry (Å, °)

*D*—H⋯*A*	*D*—H	H⋯*A*	*D*⋯*A*	*D*—H⋯*A*
N1—H1⋯O1^i^	0.916 (19)	2.153 (19)	3.0262 (15)	159.0 (15)

Symmetry code: (i) 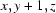.

**Fig. 1.**
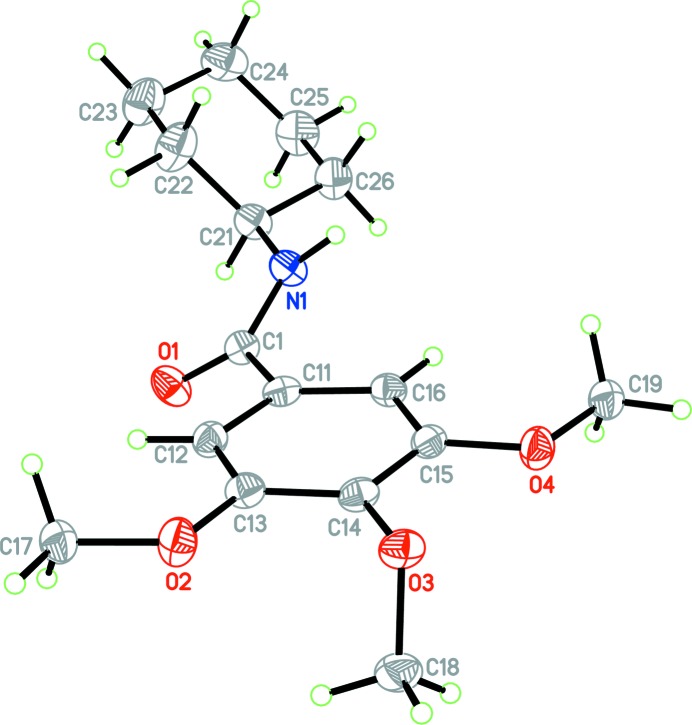
Molecular structure of title compound. Displacement ellipsoids are drawn at the 50% probability level.

**Fig. 2.**
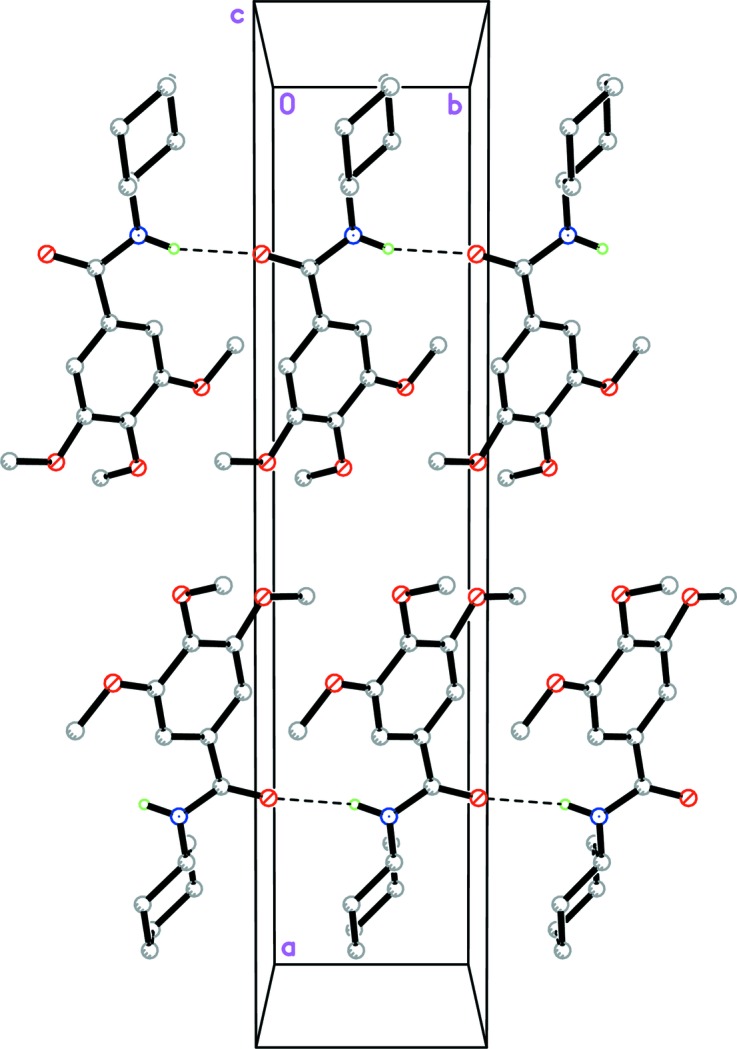
Crystal packing viewed along [001] with intermolecular hydrogen bonds indicated as dashed lines. H-atoms not involved in hydrogen bonding are omitted.

## supplementary crystallographic information

### Comment

N-substituted benzamides are well known anticancer compounds and the mechanism of action for N-substituted benzamide-induced apoptosis has been studied, using declopramide as a lead compound (Olsson *et al.*, 2002). N-substituted benzamides inhibit the activity of nuclear factor- B and nuclear factor of activated T cells activity while inducing activator protein 1 activity in T lymphocytes (Lindgren *et al.*, 2001). Heterocyclic analogs of benzanilide derivatives are potassium channel activators (Calderone *et al.*, 2006). *N*-Alkylated 2-nitrobenzamides are intermediates in the synthesis of dibenzo[b,e][1,4]diazepines (Zhichkin *et al.*, 2007) and *N*-Acyl-2-nitrobenzamides are precursors of 2,3-disubstitued 3*H*-quinazoline-4-ones (Beccalli *et al.*, 2005).

The two *m*-methoxy groups the title compound are almost coplanar with the aromatic ring [CH_3_—O—C—C -1.51 (18)° and 0.73 (19)°] whereas the methoxy group in *para* position is bent out of the plane of the aromatic ring [CH_3_—O—C—C 75.33 (15)°]. The cyclohexyl ring adops a chair conformation. The molecules are connected by N—H···O hydrogen bonds to chains running along the *b* axis.

### Experimental

3,4,5-Trimethoxybenzoyl chloride (1 mmol) in CHCl_3_ was treated with cyclohexylamine (3.5 mmol) under a nitrogen atmosphere at reflux for 3 h. Upon cooling, the reaction mixture was diluted with CHCl_3_ and washed consecutively with 1 *M* aq HCl and saturated aq NaHCO_3_. The organic layer was dried over anhydrous sodium sulfate and concentrated under reduced pressure. Crystallization of the residue in methanol afforded the title compound (78%) as colourless crystals: Anal. calcd. for C_16_H_23_N_O_4: C, 65.51; H, 9.70; N, 4.77%; found: C, 65.58; H, 9.65; N, 4.81%.

### Refinement

Hydrogen atoms were located in difference syntheses, but those bonded to C were refined at idealized positions using a riding model with C–H = 0.95–1.00 Å) and with *U*_iso_(H) = 1.2*U*_eq_(C) or *U*_iso_(H) = 1.5*U*_eq_(C_methyl_). The methyl groups were allowed to rotate but not to tip. The H atom bonded to N was freely refined.

### Crystal data

**Table:**

C_16_H_23_NO_4_	*F*(000) = 632
*M**_r_* = 293.35	*D*_x_ = 1.281 Mg m^−^^3^
Monoclinic, *P*2_1_/*c*	Mo *K*α radiation, λ = 0.71073 Å
Hall symbol: -P 2ybc	Cell parameters from 6103 reflections
*a* = 23.4539 (19) Å	θ = 3.4–26.0°
*b* = 5.2145 (6) Å	µ = 0.09 mm^−^^1^
*c* = 12.4559 (10) Å	*T* = 173 K
β = 92.886 (6)°	Block, colourless
*V* = 1521.4 (2) Å^3^	0.37 × 0.37 × 0.33 mm
*Z* = 4	

### Data collection

**Table:**

Stoe IPDSII two-circle diffractometer	2360 reflections with *I* > 2σ(*I*)
Radiation source: fine-focus sealed tube	*R*_int_ = 0.062
graphite	θ_max_ = 25.6°, θ_min_ = 3.4°
ω scans	*h* = −28→23
6868 measured reflections	*k* = −5→6
2823 independent reflections	*l* = −15→15

### Refinement

**Table:**

Refinement on *F*^2^	Secondary atom site location: difference Fourier map
Least-squares matrix: full	Hydrogen site location: inferred from neighbouring sites
*R*[*F*^2^ > 2σ(*F*^2^)] = 0.040	H atoms treated by a mixture of independent and constrained refinement
*wR*(*F*^2^) = 0.109	*w* = 1/[σ^2^(*F*_o_^2^) + (0.0651*P*)^2^ + 0.0617*P*] where *P* = (*F*_o_^2^ + 2*F*_c_^2^)/3
*S* = 1.05	(Δ/σ)_max_ < 0.001
2823 reflections	Δρ_max_ = 0.27 e Å^−^^3^
198 parameters	Δρ_min_ = −0.22 e Å^−^^3^
0 restraints	Extinction correction: *SHELXL97* (Sheldrick, 2008), Fc^*^=kFc[1+0.001xFc^2^λ^3^/sin(2θ)]^-1/4^
Primary atom site location: structure-invariant direct methods	Extinction coefficient: 0.023 (3)

### Special details

**Table:**

Geometry. All e.s.d.'s (except the e.s.d. in the dihedral angle between two l.s. planes) are estimated using the full covariance matrix. The cell e.s.d.'s are taken into account individually in the estimation of e.s.d.'s in distances, angles and torsion angles; correlations between e.s.d.'s in cell parameters are only used when they are defined by crystal symmetry. An approximate (isotropic) treatment of cell e.s.d.'s is used for estimating e.s.d.'s involving l.s. planes.
Refinement. Refinement of *F*^2^ against ALL reflections. The weighted *R*-factor *wR* and goodness of fit *S* are based on *F*^2^, conventional *R*-factors *R* are based on *F*, with *F* set to zero for negative *F*^2^. The threshold expression of *F*^2^ > σ(*F*^2^) is used only for calculating *R*-factors(gt) *etc*. and is not relevant to the choice of reflections for refinement. *R*-factors based on *F*^2^ are statistically about twice as large as those based on *F*, and *R*- factors based on ALL data will be even larger.

### Fractional atomic coordinates and isotropic or equivalent isotropic displacement parameters (Å^2^)

**Table:**

	*x*	*y*	*z*	*U*_iso_*/*U*_eq_	
N1	0.19554 (5)	0.4159 (2)	0.53398 (9)	0.0237 (3)	
H1	0.2089 (8)	0.579 (4)	0.5241 (13)	0.034 (4)*	
O1	0.21507 (4)	−0.01048 (18)	0.53804 (8)	0.0289 (3)	
O2	0.42937 (4)	0.00520 (19)	0.45580 (7)	0.0279 (3)	
O3	0.43346 (4)	0.36344 (18)	0.30218 (7)	0.0242 (2)	
O4	0.34531 (4)	0.66623 (18)	0.25120 (7)	0.0255 (2)	
C1	0.22841 (6)	0.2119 (2)	0.51372 (10)	0.0211 (3)	
C11	0.28301 (5)	0.2637 (2)	0.45950 (9)	0.0198 (3)	
C12	0.32948 (6)	0.1047 (2)	0.48557 (9)	0.0213 (3)	
H12	0.3265	−0.0265	0.5379	0.026*	
C13	0.38033 (6)	0.1395 (3)	0.43442 (9)	0.0211 (3)	
C14	0.38357 (5)	0.3267 (2)	0.35373 (9)	0.0202 (3)	
C15	0.33712 (6)	0.4871 (2)	0.32918 (9)	0.0198 (3)	
C16	0.28646 (6)	0.4586 (2)	0.38262 (9)	0.0201 (3)	
H16	0.2550	0.5693	0.3671	0.024*	
C17	0.42929 (6)	−0.1910 (3)	0.53660 (10)	0.0268 (3)	
H17A	0.4199	−0.1149	0.6055	0.040*	
H17B	0.4671	−0.2707	0.5438	0.040*	
H17C	0.4008	−0.3214	0.5157	0.040*	
C18	0.44384 (6)	0.1648 (3)	0.22488 (11)	0.0294 (3)	
H18A	0.4411	−0.0034	0.2593	0.044*	
H18B	0.4821	0.1863	0.1981	0.044*	
H18C	0.4154	0.1767	0.1647	0.044*	
C19	0.29830 (6)	0.8303 (3)	0.22110 (10)	0.0258 (3)	
H19A	0.2659	0.7263	0.1939	0.039*	
H19B	0.3095	0.9489	0.1649	0.039*	
H19C	0.2873	0.9282	0.2839	0.039*	
C21	0.14322 (6)	0.3901 (3)	0.59287 (11)	0.0247 (3)	
H21	0.1261	0.2185	0.5755	0.030*	
C22	0.15657 (7)	0.4025 (4)	0.71420 (11)	0.0376 (4)	
H22A	0.1758	0.5670	0.7323	0.045*	
H22B	0.1831	0.2617	0.7357	0.045*	
C23	0.10231 (7)	0.3799 (4)	0.77699 (12)	0.0404 (4)	
H23A	0.0851	0.2082	0.7649	0.048*	
H23B	0.1122	0.3984	0.8548	0.048*	
C24	0.05945 (7)	0.5845 (3)	0.74171 (12)	0.0372 (4)	
H24A	0.0750	0.7557	0.7612	0.045*	
H24B	0.0239	0.5599	0.7800	0.045*	
C25	0.04589 (7)	0.5735 (4)	0.62060 (12)	0.0376 (4)	
H25A	0.0196	0.7153	0.5994	0.045*	
H25B	0.0264	0.4097	0.6023	0.045*	
C26	0.10009 (6)	0.5950 (3)	0.55750 (11)	0.0310 (3)	
H26A	0.0901	0.5757	0.4797	0.037*	
H26B	0.1173	0.7668	0.5692	0.037*	

### Atomic displacement parameters (Å^2^)

**Table:**

	*U*^11^	*U*^22^	*U*^33^	*U*^12^	*U*^13^	*U*^23^
N1	0.0193 (6)	0.0209 (6)	0.0316 (6)	−0.0013 (5)	0.0099 (4)	0.0005 (4)
O1	0.0248 (5)	0.0215 (5)	0.0413 (6)	−0.0004 (4)	0.0099 (4)	0.0034 (4)
O2	0.0198 (5)	0.0365 (6)	0.0277 (5)	0.0075 (4)	0.0047 (4)	0.0091 (4)
O3	0.0189 (5)	0.0278 (5)	0.0266 (5)	−0.0017 (4)	0.0083 (4)	−0.0024 (4)
O4	0.0250 (5)	0.0272 (5)	0.0248 (4)	0.0026 (4)	0.0065 (4)	0.0070 (4)
C1	0.0188 (6)	0.0230 (6)	0.0215 (6)	−0.0005 (5)	0.0011 (5)	−0.0006 (5)
C11	0.0184 (6)	0.0221 (6)	0.0192 (5)	−0.0026 (5)	0.0036 (5)	−0.0048 (5)
C12	0.0218 (7)	0.0225 (6)	0.0197 (6)	−0.0007 (5)	0.0030 (5)	0.0009 (5)
C13	0.0175 (6)	0.0248 (6)	0.0210 (6)	0.0015 (5)	0.0007 (5)	−0.0025 (5)
C14	0.0172 (6)	0.0240 (6)	0.0197 (6)	−0.0025 (5)	0.0039 (5)	−0.0035 (5)
C15	0.0214 (7)	0.0205 (6)	0.0178 (6)	−0.0012 (5)	0.0024 (5)	−0.0011 (4)
C16	0.0176 (6)	0.0212 (6)	0.0215 (6)	0.0018 (5)	0.0009 (5)	−0.0017 (5)
C17	0.0278 (7)	0.0269 (7)	0.0254 (6)	0.0054 (6)	−0.0003 (5)	0.0037 (5)
C18	0.0293 (8)	0.0299 (7)	0.0301 (7)	0.0031 (6)	0.0125 (6)	−0.0023 (6)
C19	0.0287 (7)	0.0255 (7)	0.0233 (6)	0.0040 (6)	0.0007 (5)	0.0033 (5)
C21	0.0190 (7)	0.0246 (7)	0.0312 (7)	−0.0017 (6)	0.0086 (5)	−0.0003 (5)
C22	0.0250 (8)	0.0572 (10)	0.0312 (8)	0.0053 (7)	0.0064 (6)	0.0073 (7)
C23	0.0338 (9)	0.0543 (10)	0.0342 (8)	0.0028 (8)	0.0124 (7)	0.0066 (7)
C24	0.0318 (8)	0.0385 (9)	0.0431 (8)	−0.0008 (7)	0.0184 (7)	−0.0058 (7)
C25	0.0218 (8)	0.0462 (9)	0.0456 (9)	0.0068 (7)	0.0085 (6)	0.0034 (7)
C26	0.0238 (8)	0.0352 (8)	0.0346 (7)	0.0050 (6)	0.0073 (6)	0.0057 (6)

### Geometric parameters (Å, °)

**Table:**

N1—C1	1.3451 (17)	C18—H18B	0.9800
N1—C21	1.4670 (16)	C18—H18C	0.9800
N1—H1	0.916 (19)	C19—H19A	0.9800
O1—C1	1.2424 (16)	C19—H19B	0.9800
O2—C13	1.3615 (16)	C19—H19C	0.9800
O2—C17	1.4353 (16)	C21—C26	1.521 (2)
O3—C14	1.3761 (15)	C21—C22	1.529 (2)
O3—C18	1.4432 (16)	C21—H21	1.0000
O4—C15	1.3681 (15)	C22—C23	1.532 (2)
O4—C19	1.4307 (17)	C22—H22A	0.9900
C1—C11	1.5020 (17)	C22—H22B	0.9900
C11—C12	1.3945 (19)	C23—C24	1.516 (2)
C11—C16	1.4015 (18)	C23—H23A	0.9900
C12—C13	1.3920 (18)	C23—H23B	0.9900
C12—H12	0.9500	C24—C25	1.527 (2)
C13—C14	1.4058 (18)	C24—H24A	0.9900
C14—C15	1.3951 (19)	C24—H24B	0.9900
C15—C16	1.3988 (18)	C25—C26	1.5320 (19)
C16—H16	0.9500	C25—H25A	0.9900
C17—H17A	0.9800	C25—H25B	0.9900
C17—H17B	0.9800	C26—H26A	0.9900
C17—H17C	0.9800	C26—H26B	0.9900
C18—H18A	0.9800		
			
C1—N1—C21	121.53 (11)	H19A—C19—H19B	109.5
C1—N1—H1	120.3 (11)	O4—C19—H19C	109.5
C21—N1—H1	117.0 (11)	H19A—C19—H19C	109.5
C13—O2—C17	118.23 (10)	H19B—C19—H19C	109.5
C14—O3—C18	112.80 (10)	N1—C21—C26	110.57 (11)
C15—O4—C19	117.42 (10)	N1—C21—C22	110.82 (11)
O1—C1—N1	122.58 (12)	C26—C21—C22	110.89 (12)
O1—C1—C11	120.55 (12)	N1—C21—H21	108.1
N1—C1—C11	116.87 (11)	C26—C21—H21	108.1
C12—C11—C16	121.26 (11)	C22—C21—H21	108.2
C12—C11—C1	117.54 (11)	C21—C22—C23	111.55 (13)
C16—C11—C1	121.16 (11)	C21—C22—H22A	109.3
C13—C12—C11	119.56 (11)	C23—C22—H22A	109.3
C13—C12—H12	120.2	C21—C22—H22B	109.3
C11—C12—H12	120.2	C23—C22—H22B	109.3
O2—C13—C12	125.32 (11)	H22A—C22—H22B	108.0
O2—C13—C14	114.92 (11)	C24—C23—C22	110.72 (13)
C12—C13—C14	119.75 (12)	C24—C23—H23A	109.5
O3—C14—C15	119.15 (11)	C22—C23—H23A	109.5
O3—C14—C13	120.54 (12)	C24—C23—H23B	109.5
C15—C14—C13	120.22 (11)	C22—C23—H23B	109.5
O4—C15—C14	115.40 (11)	H23A—C23—H23B	108.1
O4—C15—C16	124.30 (12)	C23—C24—C25	111.23 (13)
C14—C15—C16	120.29 (11)	C23—C24—H24A	109.4
C15—C16—C11	118.82 (12)	C25—C24—H24A	109.4
C15—C16—H16	120.6	C23—C24—H24B	109.4
C11—C16—H16	120.6	C25—C24—H24B	109.4
O2—C17—H17A	109.5	H24A—C24—H24B	108.0
O2—C17—H17B	109.5	C24—C25—C26	111.54 (13)
H17A—C17—H17B	109.5	C24—C25—H25A	109.3
O2—C17—H17C	109.5	C26—C25—H25A	109.3
H17A—C17—H17C	109.5	C24—C25—H25B	109.3
H17B—C17—H17C	109.5	C26—C25—H25B	109.3
O3—C18—H18A	109.5	H25A—C25—H25B	108.0
O3—C18—H18B	109.5	C21—C26—C25	110.91 (12)
H18A—C18—H18B	109.5	C21—C26—H26A	109.5
O3—C18—H18C	109.5	C25—C26—H26A	109.5
H18A—C18—H18C	109.5	C21—C26—H26B	109.5
H18B—C18—H18C	109.5	C25—C26—H26B	109.5
O4—C19—H19A	109.5	H26A—C26—H26B	108.0
O4—C19—H19B	109.5		
			
C21—N1—C1—O1	4.06 (19)	C19—O4—C15—C16	−1.51 (18)
C21—N1—C1—C11	−175.93 (11)	O3—C14—C15—O4	2.05 (17)
O1—C1—C11—C12	−32.62 (17)	C13—C14—C15—O4	178.53 (11)
N1—C1—C11—C12	147.36 (12)	O3—C14—C15—C16	−178.18 (11)
O1—C1—C11—C16	145.07 (13)	C13—C14—C15—C16	−1.70 (18)
N1—C1—C11—C16	−34.94 (17)	O4—C15—C16—C11	178.64 (11)
C16—C11—C12—C13	−0.02 (18)	C14—C15—C16—C11	−1.11 (18)
C1—C11—C12—C13	177.67 (11)	C12—C11—C16—C15	1.99 (18)
C17—O2—C13—C12	0.73 (19)	C1—C11—C16—C15	−175.62 (11)
C17—O2—C13—C14	−179.46 (11)	C1—N1—C21—C26	−150.78 (12)
C11—C12—C13—O2	177.00 (12)	C1—N1—C21—C22	85.85 (16)
C11—C12—C13—C14	−2.81 (19)	N1—C21—C22—C23	179.14 (13)
C18—O3—C14—C15	−108.20 (13)	C26—C21—C22—C23	55.95 (18)
C18—O3—C14—C13	75.33 (15)	C21—C22—C23—C24	−56.00 (19)
O2—C13—C14—O3	0.29 (17)	C22—C23—C24—C25	55.59 (19)
C12—C13—C14—O3	−179.89 (11)	C23—C24—C25—C26	−55.74 (19)
O2—C13—C14—C15	−176.15 (11)	N1—C21—C26—C25	−178.60 (12)
C12—C13—C14—C15	3.68 (19)	C22—C21—C26—C25	−55.27 (17)
C19—O4—C15—C14	178.25 (11)	C24—C25—C26—C21	55.42 (18)

### Hydrogen-bond geometry (Å, °)

**Table:**

*D*—H···*A*	*D*—H	H···*A*	*D*···*A*	*D*—H···*A*
N1—H1···O1^i^	0.916 (19)	2.153 (19)	3.0262 (15)	159.0 (15)

Symmetry codes: (i) *x*, *y*+1, *z*.
